# The FDP/FIB Ratio and Blood FDP Level May Be Related to Seizures After Fever in Young Children

**DOI:** 10.3389/fped.2020.00439

**Published:** 2020-07-31

**Authors:** Chun Li, Weining Ma, Shaoyi Li, Yajuan Zhao, Xuyang Zhao, Hua Wang

**Affiliations:** ^1^Department of Pediatrics, Shengjing Hospital of China Medical University, Shenyang, China; ^2^Department of Neurosurgery, Shengjing Hospital of China Medical University, Shenyang, China; ^3^Beijing Key Laboratory of Tumor Systems Biology, Department of Pathology, Peking-Tsinghua Center for Life Sciences, School of Basic Medical Sciences, Institute of Systems Biomedicine, Peking University Health Science Center, Beijing, China

**Keywords:** simple febrile seizures, fibrinogen degradation products, fibrinogen, fever, blood-brain barrier

## Abstract

**Objective:** To evaluate the relationship of the blood fibrinogen (FIB) degradation product (FDP) level and FDP/FIB ratio with seizure in young children with fever.

**Methods:** A total of 35 children with simple febrile seizures and 80 children with fever but no seizure were selected. First, the differences in white blood cell (WBC), platelets (PLT), prothrombin time (PT), activated partial thromboplastin time (APTT), thrombin time (TT), FIB, FDP, FDP/FIB ratio, and C-reactive protein (CRP) between 35 children with simple febrile seizures and 40 randomly selected children with fever but no seizure were retrospectively analyzed. Then, an ROC curve was used to determine the diagnostic utility of the FDP level, FDP/FIB ratio, and FDP+FDP/FIB ratio, and the best diagnostic cutoff points were selected. Finally, the diagnostic specificities of the three diagnostic indicators were verified by comparison with the results of all 80 children with fever but no seizure.

**Results:** The FDP level and FDP/FIB ratio were significantly different between the two groups (*P* < 0.0001) and there was a positive correlation between the FDP and FIB levels. Both the FDP level and FDP/FIB ratio had good diagnostic value. An FDP ≥ 2.0 mg/L and FDP/FIB ratio ≥ 0.5 had good diagnostic specificities. Combined application of an FDP ≥ 2.0 mg/L and FDP/FIB ratio ≥ 0.5 improved the diagnostic power.

**Conclusions:** The blood FDP level and FDP/FIB ratio may be related to seizures after fever, and an FDP ≥ 2.0 mg/L + FDP/FIB ratio ≥ 0.5 has good diagnostic specificity.

## Introduction

Fever is a common sign of pediatric emergency in children younger than 5 years of age, mostly due to infections. Whether fever can cause seizures, and which children with fever are at risk of seizures, are matters of great concern to pediatricians and parents. In pediatrics, febrile seizures (FS), particularly simple febrile seizures (SFS), is the most common cause of seizure (1, 2). According to the definition, FS is a type of seizure accompanied by fever that is seen in children aged 6 months to 5 years with no evidence of infection in the central nervous system or other identifiable causes of seizure (3). SFS is one subtype of FS with seizures that last for <15 min, with generalized seizure occurring only once in 24 h (4).

Our previous serum proteomics studies showed that the levels of fibrinogen (FIB)-related proteins in SFS children decreased significantly compared to children with fever but no seizure (5). In addition, recent work has shown that FIB deposition in brain tissue is closely related to the occurrence and development of multiple sclerosis, Alzheimer's disease, and other central nervous system diseases (6). FIB is produced by the liver, and it enters brain tissue through the blood–brain barrier (BBB). Accordingly, FIB deposition in brain tissue also reflects enhanced permeability of the BBB (7). Thus, the process of FIB transfer *in vivo* indicates that factors exogenous to the nervous system can also lead to neurological diseases. According to the definition of febrile seizures, seizures in children are caused by factors exogenous to the nervous system. However, it is still not clear whether fibrinogen is this exogenous factor that causes FS. According to previous studies (8, 9), the permeability of the BBB changes with brain development, and at 6 months, BBB permeability approaches adult levels, which may provide a physiological basis for FIB penetration from blood into brain tissue. To the best of our knowledge, no study has been conducted on the relationship between BBB permeability and FS. Therefore, based on the results of our previous serum proteomics studies, we wanted to further explore the differences in expression levels of FIB and related laboratory parameters for routine clinical diagnosis between the children with SFS and the children with fever but no seizure.

In this retrospective study, children with SFS (SFS group) and children with fever but no seizure (FNS group) were investigated. The blood levels of FIB, fibrinogen degradation products (FDP), white blood cell (WBC), platelet (PLT), prothrombin time (PT), activated partial thromboplastin time (APTT), thrombin time (TT), and C-reactive protein (CRP) of the children in the two groups were compared to determine the presence of any differences and which differences could be used as diagnostic indicators.

## Materials and Methods

### Patients Collected

All of the patients were from the Department of Pediatrics, Shengjing Hospital of China Medical University. There were 35 patients with SFS. SFS diagnosis was based on seizure type, duration, frequency, and elevated temperature with no definite pyrogens were found. Patients with intracranial infection, complex febrile seizures, or epilepsy were excluded from this study. The FNS group included 80 hospitalized patients with fever caused by infectious diseases such as bacterial pneumonia. The blood collection time of both groups was within 24 h after hospitalization. The age, sex, peak temperature, fever duration, WBC, PLT, PT, APTT, TT, FIB, FDP, CRP data of the two groups were reviewed and the FDP/FIB ratio calculated. This study was approved by the ethics committee of Shengjing Hospital of China Medical University.

### Research Process

**Figure 1:**
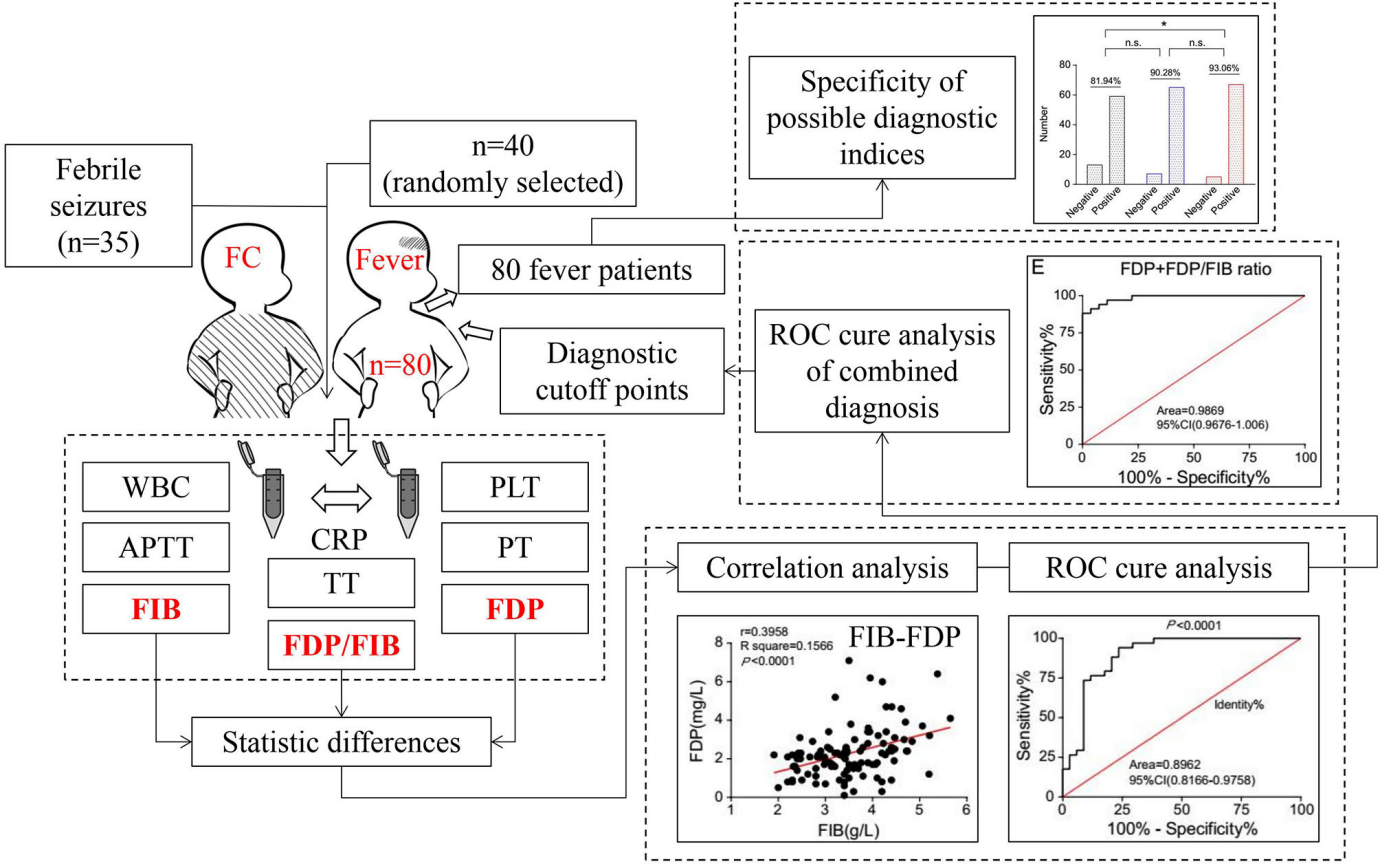
The overall scheme of this study.

In step one, the diagnostic indicators were screened and diagnostic cutoff points selected. All 35 patients with SFS were selected as the experimental group, and 40 patients with FNS were randomly selected as the control group. First, the differences in WBC, PLT, PT, APTT, TT, FIB, FDP, FDP/FIB ratio, and CRP data between the two groups were compared, and the variables with statistically significant differences were selected as diagnostic indicators. Then, the sensitivity and specificity of these diagnostic indicators were analyzed. Finally, the appropriate diagnostic cutoff points were selected. In step two, the diagnostic specificities of the diagnostic cutoff points were verified. Using the diagnostic cutoff points of the diagnostic indices obtained by the above step, the diagnostic specificities of all 80 FNS patients were evaluated (Figure 1).

### Statistical Analysis

The statistical differences in age, fever duration, peak temperature, the FDP/FIB ratio, and WBC, PLT, PT, APTT, TT, FIB, FDP, CRP levels between the SFS and FNS groups were calculated by *t*-test. Linear regression analysis was used to analyze the correlation between FDP and FIB. The correlation between CRP and FDP, FIB, FDP/FIB ratio was analyzed using the linear regression analysis. The sensitivities and specificities of the diagnostic indicators were analyzed using the area under the ROC curve (AUC) and compared using the chi-square test for independence. SPSS 17.0 software was used for statistical analysis, and *P* < 0.05 was considered statistically significant.

## Results

### Patients Characteristics

There were 35 patients with SFS, and other 80 patients with FNS. The seizure type in SFS group was tonic-clonic with a duration <15 min, the EEG and MRI examination were normal in all patients (Table 1). The FNS group included 39 males and 41 females under the age of 60 months. The causes of fever in the FNS group were infectious diseases such as pneumonia (Table 2). There were no significant differences in age, peak temperature, or fever duration between the two groups (*P* > 0.05).

**Table 1:** The demographic of the SFS cases.

The total number of cases		35
Sex	Male	27
	Female	8
Age (months)		8–60 (AVE 31 ± 14.8)
Peak temperature (^°^C)		38.5–41.2 (AVE 39.7 ± 0.7)
Fever duration (days)		1–10 (AVE 2.3 ± 1.8)
Convulsion type		Tonic-clonic
MRI examination		Normal
EEG examination		Normal

**Table 2:** The demographic of the FNS cases.

The total number of cases		80
Sex	Male	39
	Female	41
Age (months)		6–60 (AVE 36.4 ± 14.0)
Peak temperature (^°^C)		38.5–41.0 (AVE 39.4 ± 0.6)
Fever duration (days)		1–6 (AVE 2.9 ± 1.2)
	P	28
Pathogeny	MPIP	15
	EBIP	1
	HSVP	2
	P+Suppurative tonsillitis	2
	P+Agranulocytosis	1
	P+Urinary tract infection	1
	P+Sepsis	2
	P+MPI+EBI	1
	P+Sepsis+Suppurative tonsillitis	2
	EBI+MPI	2
	Laryngitis	1
	Suppurative tonsillitis	1
	Urinary tract infection	1
	Sepsis	12
	Sepsis+MPI	1
	Sepsis+Cervical lymphadenitis	1
	Sepsis+Herpetic pharyngitis	1
	Bronchitis	1
	Bronchitis+MPI	1
	Bronchitis+Suppurative tonsillitis	1
	Bronchitis+Sepsis+MPI	1

*P, bacterial pneumonia; MPIP, mycoplasma pneumoniae infection pneumonia; EBIP, epstein-barr virus infection pneumonia; HSVP, Herpes simplex virus infection pneumonia*.

### Results of Blood Test Indicators in the Screening Step

**Figure 2:**
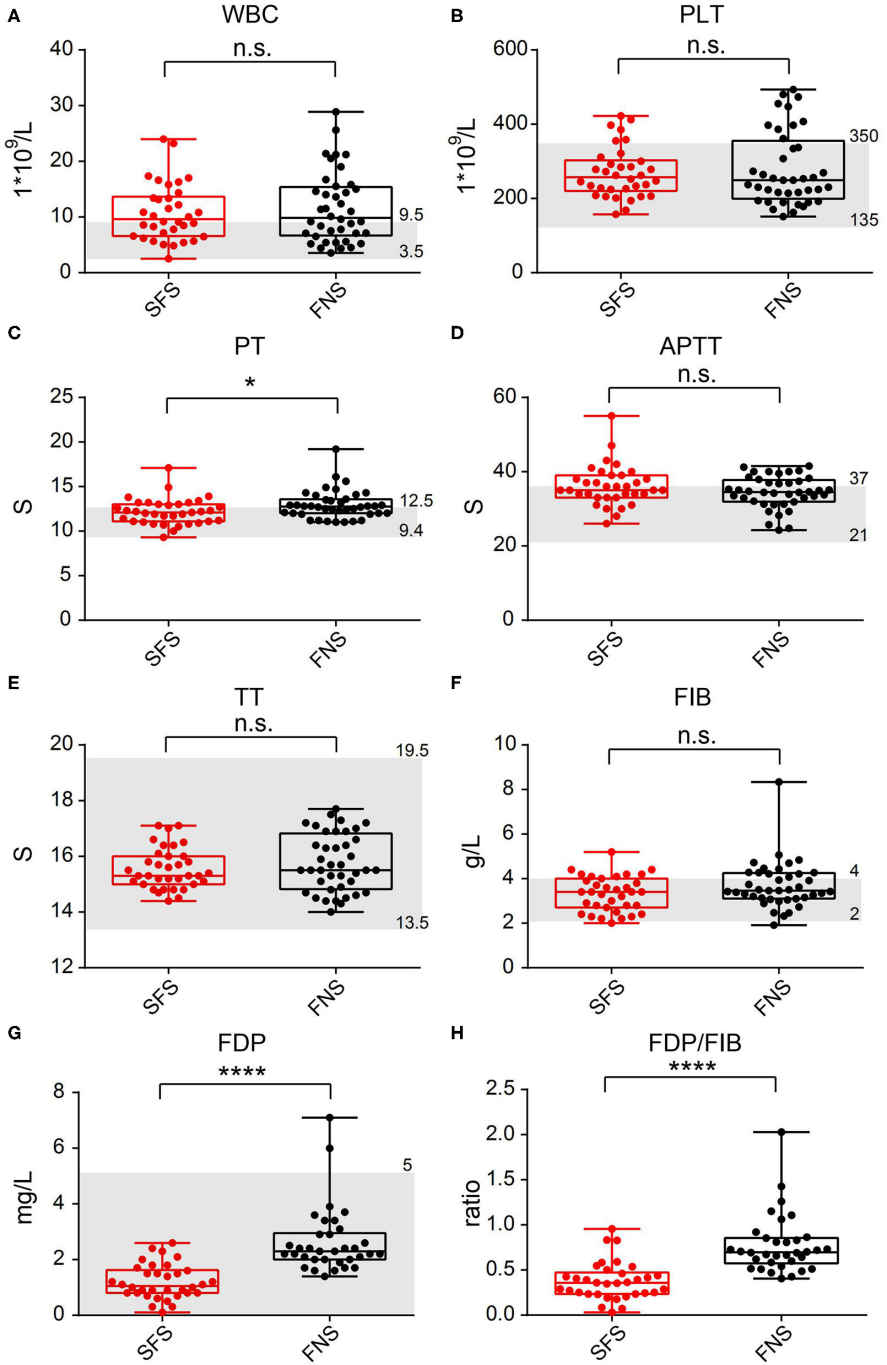
Comparison of laboratory test indicators between the SFS and FNS groups. **(A)** WBC. **(B)** PLT. **(C)** PT. **(D)** APTT. **(E)** TT. **(F)** FIB. **(G)** FDP. **(H)** FDP/FIB. n.s.: not significant; *P < 0.05; ****P < 0.0001. The gray area represents the clinical normal range.

In the SFS group, the average WBC count was 10.77 × 10^9^/L (ranged from 2.52 × 10^9^/L to 23.99 × 10^9^/L); PLT was 269.94 × 10^9^/L (ranged from 157.00 × 10^9^/L to 422.00 × 10^9^/L); PT was 12.17 s (ranged from 9.30 to 17.10 s); APTT was 36.17 s (ranged from 26.00 to 55.00 s); TT was 15.56 s (ranged from 14.40 to 17.10 s); FIB was 3.32 g/L (ranged from 2.00 to 5.20 g/L); FDP was 1.20 mg/L (ranged from 0.10 to 2.60 mg/L); and the FDP/FIB ratio was 0.19 (ranged from 0.03 to 0.95). Among the 40 patients in the FNS group, the average WBC count was 11.64 × 10^9^/L (ranged from 3.56 × 10^9^/L to 28.90 × 10^9^/L); PLT was 279.78 × 10^9^/L (ranged from 151.00 × 10^9^/L to 493.00 × 10^9^/L); PT was 12.95 s (ranged from 11.00 to 19.20 s); APTT was 34.44 s (ranged from 24.30 to 41.50 s); TT was 15.76 s (ranged from 14.00 to 17.70 s); FIB was 3.69 g/L (ranged from 1.91 to 8.34 g/L); FDP was 2.64 mg/L (ranged from 1.40 to 7.10 mg/L); and the FDP/FIB ratio was 0.66 (ranged from 0.40 to 2.03). The differences in the FDP level and FDP/FIB ratio between the two groups were statistically significant (*P* < 0.0001), whereas there was no statistically significant difference in FIB between the two groups (Figure 2). There was also statistical difference in the PT level between the two groups in this step, however, when all 80 patients in the FNS group were counted, there was no statistical difference between the two groups.

### CRP Was Significantly Different Between the Two Groups and Positively Correlated With FDP, FIB, and FDP/FIB Ratio

**Figure 3:**
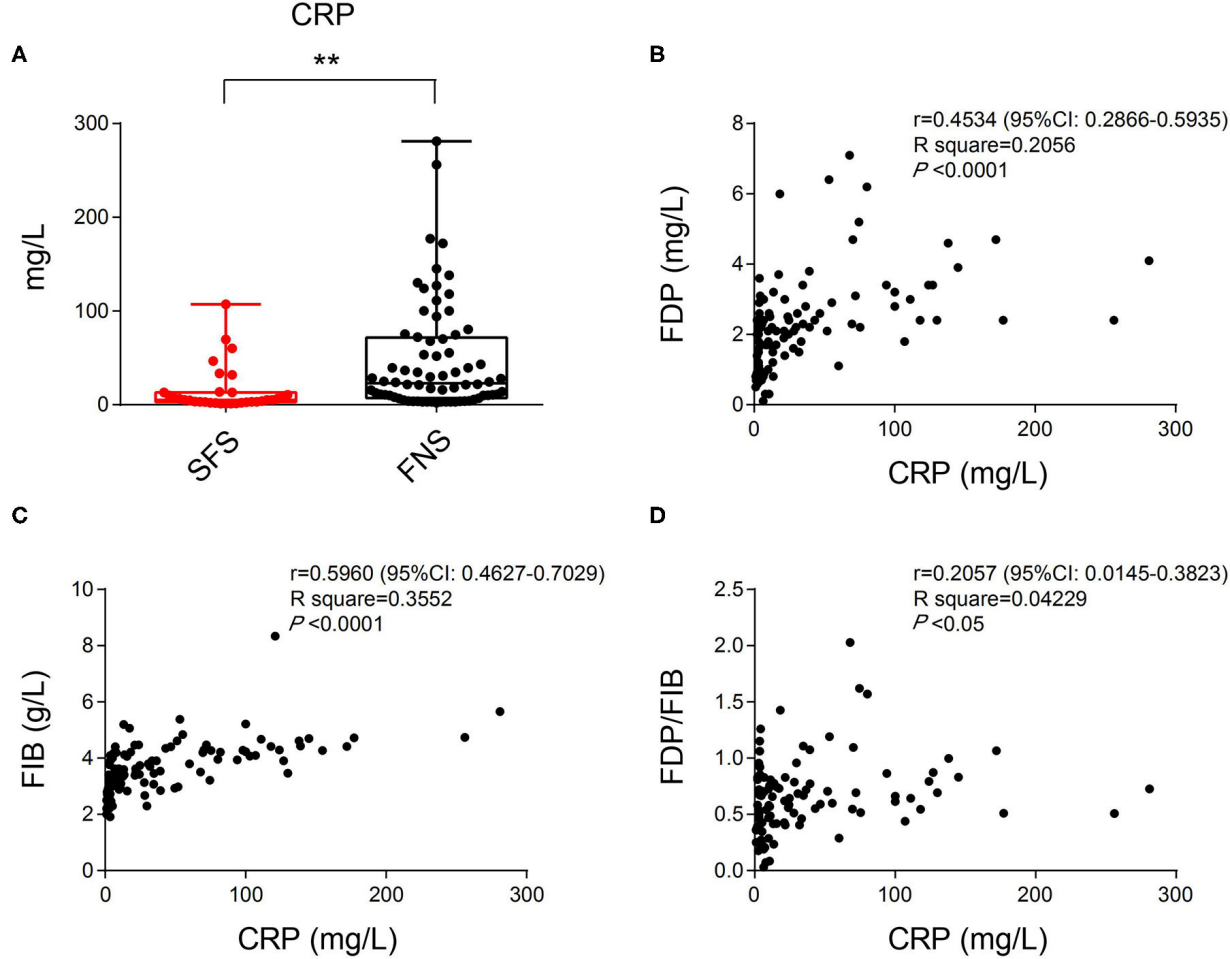
**(A)** The difference in CRP index between the SFS and FNS groups. **(B)** Correlation analysis of CRP and FDP levels showing their positive correlation. **(C)** Correlation analysis of CRP and FIB levels showing their positive correlation. **(D)** Correlation analysis of CRP and FDP/FIB ratios showing their positive correlation. **P < 0.01.

In the SFS group, the average CRP index was 14.83 mg/L (ranged from 1.05 to 69.50 mg/L), and among the 80 patients in the FNS group, the average was 47.53 mg/L (ranged from 2.00 to 281.00 mg/L). The differences in the CRP level between the two groups were statistically significant (*P* < 0.01) (Figure 3A). Correlation analysis revealed positive correlation between CRP and FDP, FIB, FDP/FIB levels (Figures 3B–D). The differences of FDP, FIB, and FDP/FIB ratio between the two groups may be dramatically influenced by patients' inflammatory state.

### Both the FDP Level and FDP/FIB Ratio Have Good Sensitivity and Specificity as Diagnostic Indices

**Figure 4:**
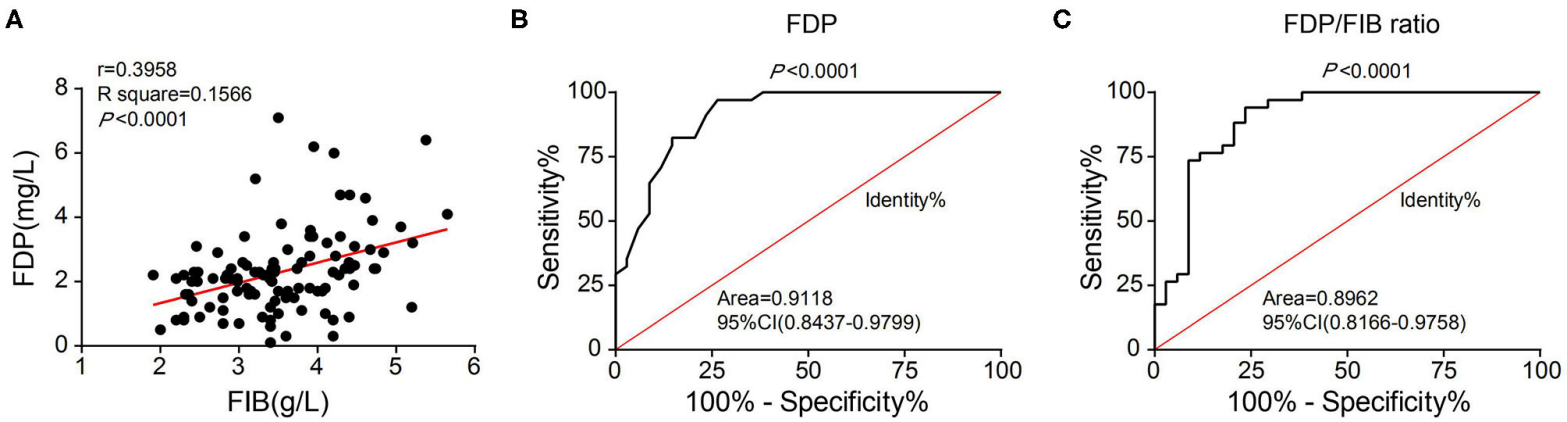
**(A)** Correlation analysis of FIB and FDP levels showing their positive correlation. **(B)** ROC curve of FDP as a diagnostic index. **(C)** ROC curve of the FDP/FIB ratio as a diagnostic index.

Although the difference in the FIB index between the two groups was not statistically significant, the average FIB was lower in the SFS group than in the FNS group. Correlation analysis revealed a positive correlation between FIB and FDP levels (*r* = 0.3958) (Figure 4A). The FDP level and FDP/FIB ratio were significantly lower in the SFS group than in the FNS group. With the ROC curve plotted and the AUC calculated, the AUC was 0.9118 (95% CI 0.8437–0.9779) when FDP was used as the diagnostic index (Figure 4B) and 0.8962 (95% CI 0.8166–0.9758) when the FDP/FIB ratio was used (Figure 4C). Both diagnostic indices had good sensitivity and specificity.

### Diagnostic Cutoff Points of the FDP Level and FDP/FIB Ratio

**Figure 5:**
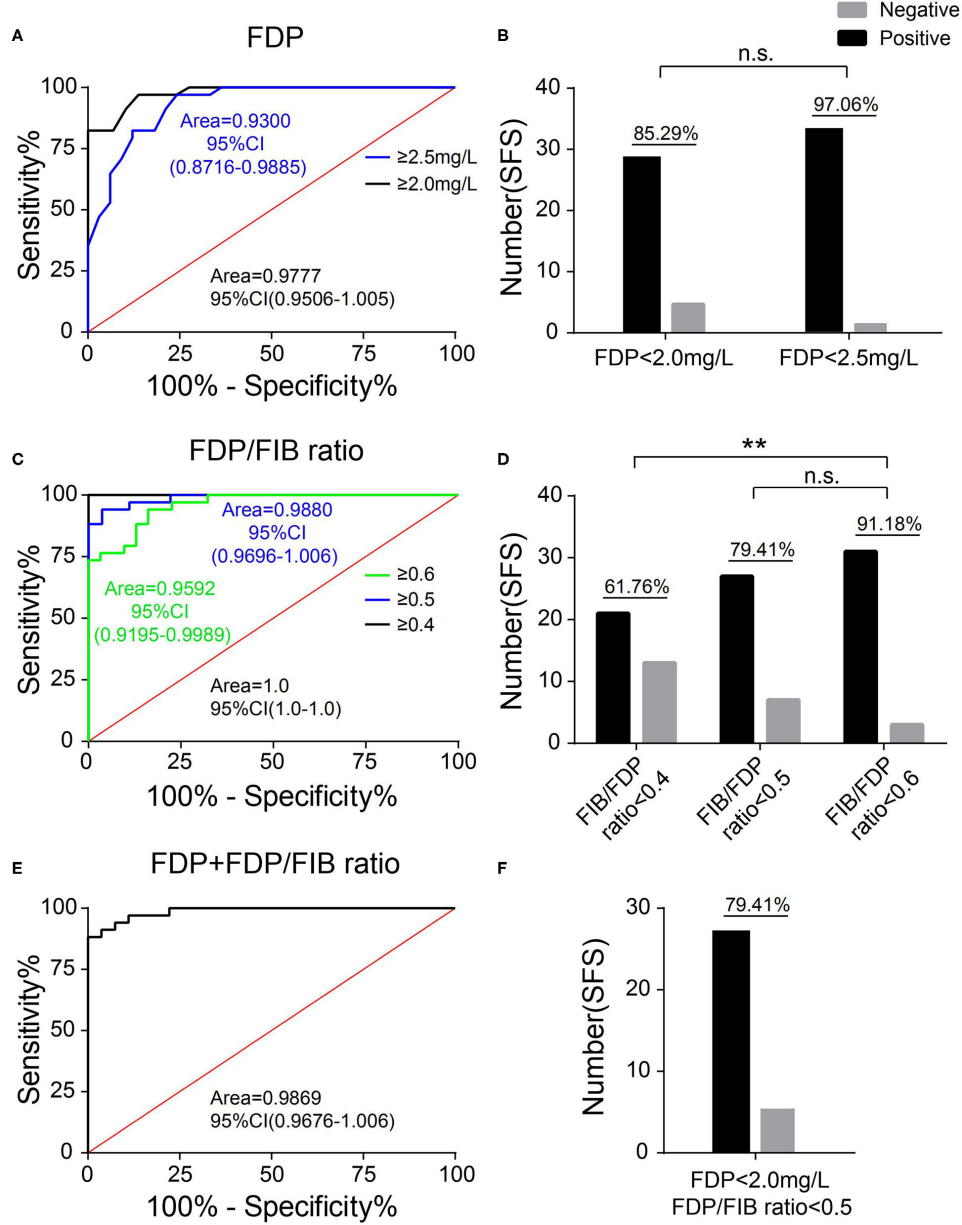
Selection of the diagnostic cutoff point. **(A)** The ROC curves of FDP ≥ 2.0 mg/L and FDP ≥ 2.5 mg/L as diagnostic cutoff points. **(B)** The proportion of patients in the SFC group with an FDP <2.0 mg/L and FDP <2.5 mg/L. **(C)** The ROC curves of an FDP/FIB ≥ 0.4, FDP/FIB ≥ 0.5, and FDP/FIB ≥ 0.6 as diagnostic cutoff points. **(D)** The proportion of patients in the SFC group with an FDP/FIB <0.4, FDP/FIB <0.5, and FDP/FIB <0.6. **(E)** The ROC curve of FDP ≥ 2.0 mg/L + FDP/FIB ≥ 0.5 as the combined diagnostic cutoff point. **(F)** The proportion of patients in the SFS group with an FDP <2.0 mg/L and FDP/FIB <0.5. n.s.: not significant; **P < 0.01.

When FDP was used as the diagnostic index, an FDP ≥ 2.0 mg/L or FDP ≥ 2.5 mg/L was used as the diagnostic cutoff point. The patients in the SFS group below these two cutoff points were used to draw the identity line, and the ROC curve was plotted and the AUC calculated with the patients in the FNS group. When an FDP ≥ 2.0 mg/L was used as the diagnostic cutoff point, the AUC was 0.9777 (95% CI 0.9506–1.005) and 85.29% of patients in the SFS group were below the cutoff point. When an FDP ≥ 2.5 mg/L was used as the diagnostic cutoff point, the AUC was 0.9300 (95% CI 0.8716–0.9885) and 97.06% of patients in the SFS group were below the cutoff point (Figures 5A,B).

When the FDP/FIB ratio was used as the diagnostic index, FDP/FIB ratios ≥ 0.4, ≥ 0.5, and ≥ 0.6 were used as diagnostic cutoff points. The patients in the SFS group below the above three cutoff points were used to draw the identity line, and the ROC curve was plotted and the AUC calculated for the patients in the FNS group. When an FDP/FIB ratio ≥ 0.4 was used as the diagnostic cutoff point, the AUC was 1.0 (95% CI 1.0–1.0) and 61.76% of the patients in the SFS group were below the cutoff point. When an FDP/FIB ratio ≥ 0.5 was used as the diagnostic cutoff point, the AUC was 0.9880 (95% CI 0.9696–1.006) and 79.41% of the patients in the SFC group were below the cutoff point. When FDP/FIB ratio ≥ 0.6 was used as the diagnostic cutoff point, the AUC was 0.9592 (95% CI 0.9195–0.9989) and 91.18% of the patients in the SFS group were below the cutoff point. The difference in the proportion of the SFS group between an FDP/FIB ratio <0.6 and FDP/FIB ratio <0.4 was statistically significant (*P* < 0.001), unlike the difference between FDP/FIB ratios < 0.6 and < 0.5 (*P* > 0.05) (Figures 5C,D).

The above results indicate that a diagnostic cutoff point of FDP ≥ 2.0 mg/L and an FDP/FIB ratio ≥ 0.5 has both good specificity and good sensitivity. When FDP ≥ 2.0 mg/L and an FDP/FIB ratio ≥ 0.5 were combined, the patients in the SFS group that simultaneously met the above two indicators (i.e., were below the cutoff point) were used to draw the identity line. At this point, the AUC was 0.9869 (95% CI 0.9676–1.006) and the patients in the SFS group below the cutoff point accounted for 79.41% (Figures 5E,F). The results show that combined diagnosis can improve the specificity vs. FDP alone.

### Differences in the Diagnostic Specificity of the FDP Level, the FDP/FIB Ratio, and FDP+FDP/FIB Ratio

**Figure 6:**
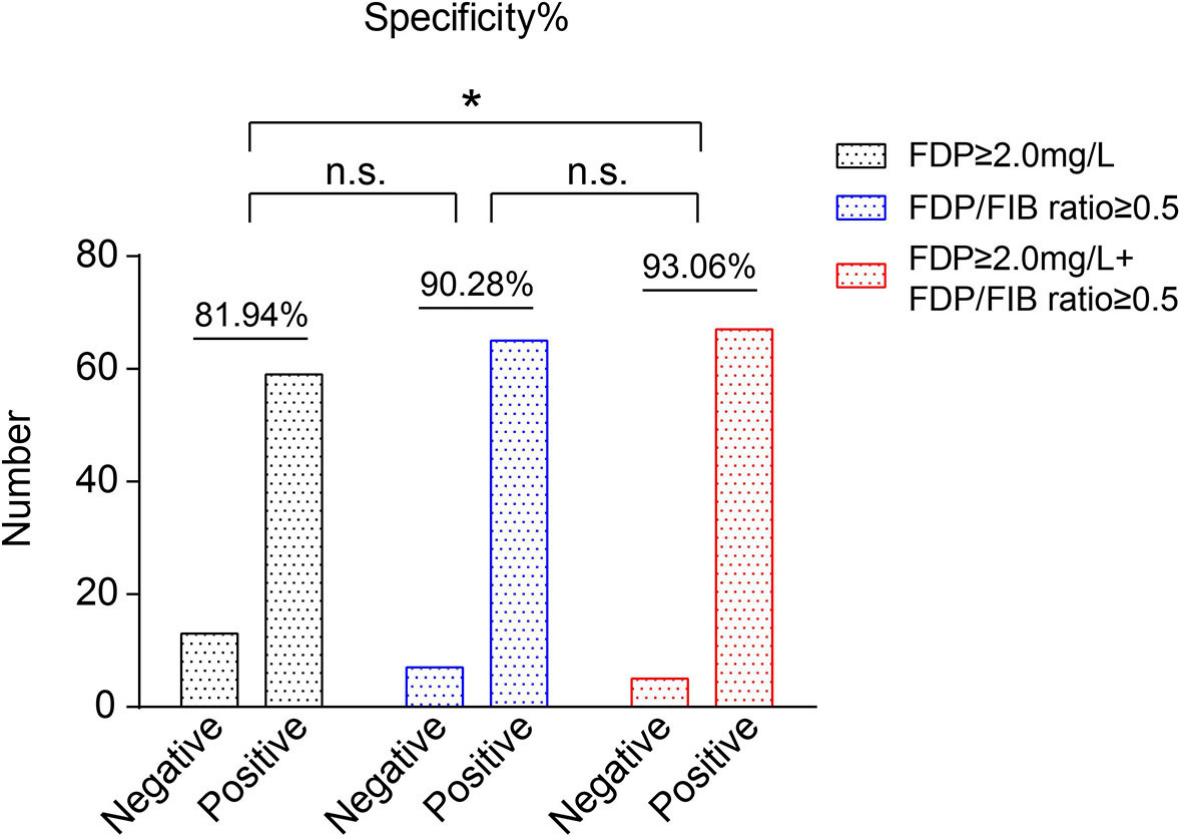
The specificities of FDP ≥ 2.0 mg/L, an FDP/FIB ≥ 0.5, and FDP ≥ 2.0 mg/L + FDP/FIB ≥ 0.5 as the diagnostic cutoff points. *P < 0.05.

The diagnostic specificities of all 80 FNS patients were compared according to FDP ≥ 2.0 mg/L, an FDP/FIB ratio ≥ 0.5, and FDP ≥ 2.0 mg/L + FDP/FIB ≥ 0.5. When FDP ≥ 2.0 mg/L alone was used as the diagnostic cutoff point, the specificity was 81.94%. When an FDP/FIB ratio ≥ 0.5 alone was used as the diagnostic cutoff point, the specificity was 90.28%. When FDP ≥ 2.0 mg/L + FDP/FIB ≥ 0.5 was used as the combined diagnostic cutoff point, the specificity was 93.06%, which was significantly higher than that of FDP ≥ 2.0 mg/L alone (*P* < 0.05) (Figure 6). The results thus showed that the combination of FDP+FDP/FIB ratio can improve the diagnostic utility.

## Discussion

### Changes in FIB and FDP Levels May Be Exogenous Nervous System Factors Leading to SFS

In this study, by comparing the differences in the levels of FIB and FDP and other laboratory indicators in the blood of patients with SFS and fever without seizure, we found that FIB was slightly lower and FDP significantly lower in the blood of children with SFS. According to the definition of SFS, the seizures are caused by exogenous factors in the nervous system, suggesting that some exogenous pathogenic factors are essential for the pathogenesis of SFS. FIB is a large protein (340 kDa) that is produced by the liver and secreted into the blood to participate in coagulation (10). However, recent studies have shown that coagulation factors such as FIB are not only involved in coagulation, but also closely related to the occurrence and development of some neurological diseases (11), such as multiple sclerosis (12) and Alzheimer disease (13). Through mechanistic studies, it was found that the occurrence of nervous system diseases such as Alzheimer disease was associated with the deposition of FIB and fibrin in brain tissues (14, 15) and that FIB and fibrin had multiple sites of action in nerve cells (16, 17) and mediated a series of inflammatory and degenerative reactions in neurons and glial cells (6). FIB enters the nervous system through blood–brain barrier leakage (7), and a high level of FIB in tissues is closely related to Alzheimer disease severity (18), indicating that enhanced permeability of the blood–brain barrier plays a key role in the occurrence of multiple sclerosis, Alzheimer disease, and other neurological diseases (19, 20).

Through previous studies, it can be concluded that there are two main ways by which FIB can cause nervous system diseases. First, a highly permeable blood–brain barrier allows large molecules such as FIB to pass. Second, FIB can act on nerve cells and mediate a series of subsequent pathogenic processes. Thus, in the pathogenesis of FS, in which of the above two conditions does FIB play a role? SFS occurs in children younger than 5 years of age due to their immature nervous system and developing BBB. Studies have shown that there are differences in the amount of constituent proteins in the BBB according to age (21), suggesting that there may be differences in BBB permeability at different ages. In addition, factors such as infection can increase the permeability of the BBB (22). Some previous studies (23, 24) have shown a significant increase in the expression of FIB in cerebrospinal fluid from diseases such as multiple sclerosis and meningitis. Our results suggest that the overall FIB level of the SFS group was decreased compared with that of the FNS group, with the FDP of its metabolite significantly decreased in the SFS group and the FDP/FIB ratio significantly decreased. We speculated that, due to the effect of an extra-nervous system infection and the developing BBB, FIB is transferred from the blood to the nervous system through the BBB to mediate the subsequent transient inflammatory response of the nervous system, leading to seizures. However, the lack of CSF testing in our cases is a limitation of this study. Therefore, further cerebrospinal fluid studies are needed in the future.

### The FDP/FIB Ratio as a Diagnostic Index May Be More Consistent With the Possible Pathogenic Process of FIB Transfer

Our study found that, although the FDP level and FDP/FIB ratio were significantly lower in the SFS group than in the FNS group, the clot-related results of the two groups were basically within the normal range while the pure FIB and FDP indicators were within the range of normal clotting function detection indicators. As our speculation, SFS may be caused by the transfer of blood FIB to brain tissue, indicating that the transfer process is increased and its catabolic conversion to FDP weakened. The application of the FDP/FIB ratio may be better reflect the catabolic metabolism ratio and the lateral reaction to the FIB transfer process. Of course, further studies about the pathogenesis of FS and how FIB participates in the pathological mechanism of FS at the cellular and molecular levels are required to identify better diagnostic indicators.

### The Inflammatory State May Also Influence the Circulating Levels of FIB and FDP

Previous studies (25, 26) have shown that inflammatory factors, such as interleukin-6, regulate the synthesis of FIB. Our findings also suggest a correlation between the patient's inflammatory state and blood levels of FDP and FIB. In the FNS group, CRP levels were significantly higher than those in the SFS group, so the high expression of FDP and FIB may be due to inflammatory factors. However, our previous sero-proteomic study found that FIB expression was at a lower level in SFS children than in healthy children (5). Therefore, whether the changes in FDP and FIB in the blood are due to the increased intracranial migration of FIB, the inflammatory state, or a combination of these two factors in the pathogenesis of SFS remains to be further investigated.

## Conclusion

Our study found that the FDP level and FDP/FIB ratio were significantly decreased in SFS patients. The FDP level in blood and the FDP/FIB ratio are related to seizures after fever in young children. When FDP ≥ 2.0 mg/L + FDP/FIB ratio ≥ 0.5 is used as the combined diagnostic cutoff point, the diagnostic specificity is good.

## Data Availability Statement

The raw data supporting the conclusions of this article will be made available by the authors, without undue reservation.

## Ethics Statement

The studies involving human participants were reviewed and approved by Ethics and the Human Research Review Committee of Shengjing Hospital affiliated to China Medical University. Written informed consent to participate in this study was provided by the participants' legal guardian/next of kin. Written informed consent was obtained from the minor(s)' legal guardian/next of kin for the publication of any potentially identifiable images or data included in this article.

## Author Contributions

All authors were involved in the study design, interpretation of the results, reviewing, approval of the manuscript, and in the decision to submit the article for publication. All authors also confirm accountability for the accuracy and integrity of the work.

## Conflict of Interest

The authors declare that the research was conducted in the absence of any commercial or financial relationships that could be construed as a potential conflict of interest.

## Abbreviations

FIB: fibrinogen
FDP: fibrinogen degradation product
WBC: white blood cell
PLT: platelets
PT: prothrombin time
APTT: activated partial thromboplastin time
TT: thrombin time
CRP: C-reactive protein
FS: febrile seizures
SFS: simple febrile seizures
BBB: blood–brain barrier.
